# Determinants of maternal high-risk fertility behaviors and its correlation with child stunting and anemia in the East Africa region: A pooled analysis of nine East African countries

**DOI:** 10.1371/journal.pone.0253736

**Published:** 2021-06-30

**Authors:** Koku Sisay Tamirat, Getayeneh Antehunegn Tesema, Zemenu Tadesse Tessema

**Affiliations:** Department of Epidemiology and Biostatistics, Institute of Public Health, College of Medicine and Health Sciences, University of Gondar, Gondar, Ethiopia; University of Mississippi Medical Center, UNITED STATES

## Abstract

**Background:**

In low-income nations, high-risk fertility behavior is a prevalent public health concern that can be ascribed to unmet family planning needs, child marriage, and a weak health system. As a result, this study aimed to determine the factors that influence high-risk fertility behavior and its impact on child stunting and anemia.

**Method:**

This study relied on secondary data sources from recent demography and health surveys of nine east African countries. Relevant data were extracted from Kids Record (KR) files and appended for the final analysis; 31,873 mother-child pairs were included in the final analysis. The mixed-effect logistic regression model (fixed and random effects) was used to describe the determinants of high-risk fertility behavior (HRFB) and its correlation with child stunting and anemia.

**Result:**

According to the pooled study about 57.6% (95% CI: 57.7 to 58.2) of women had at least one high-risk fertility behavior, with major disparities found across countries and women’s residences. Women who lived in rural areas, had healthcare access challenges, had a history of abortion, lived in better socio-economic conditions, and had antenatal care follow-up were more likely to engage in high-risk fertility practices. Consequently, Young maternal age at first birth (<18), narrow birth intervals, and high birth orders were HRFBs associated with an increased occurrences of child stunting and anemia.

**Conclusion:**

This study revealed that the magnitude of high-risk fertility behavior was higher in east Africa region. The finding of this study underscores that interventions focused on health education and behavioral change of women, and improvement of maternal healthcare access would be helpful to avert risky fertility behaviors. In brief, encouraging contraceptive utilization and creating awareness about birth spacing among reproductive-age women would be more helpful. Meanwhile, frequent nutritional screening and early intervention of children born from women who had high-risk fertility characteristics are mandatory to reduce the burden of chronic malnutrition.

## Introduction

Rapid population growth has been observed in developing countries including Sub-Saharan African countries, with an estimated population of 1.2 billion by 2025 [1]. The total fertility rate is declining globally, but it is decreasing more slowly in SSA, with a total fertility rate of 4.69 children per woman in 2018, down from 5.37 in 2008. Despite declining natural resources, a lack of infrastructures such as housing, schools, and health facilities, and increased unemployment, most African countries lack a demography and population policy to control or monitor fertility rates [2].

Women’s high-risk fertility habits, which are defined by narrow birth intervals, high birth order, and younger maternal age at birth, have been linked to negative health outcomes for both the mother and the child [3–5]. Due to increased family planning use, expansion of women’s education, and economic trends, pooled decomposition analysis revealed that high-risk fertility behavior was decreased over decades. High-risk fertility behavior (HRFB) is linked to an increased risk of infant mortality, chronic malnutrition, and adverse birth outcomes such as stillbirth, prematurity, and low birth weight, according to research findings [6–11]. The nations of the East African region share many of the same socio-demographic and cultural characteristics. Maternal and child mortality remains high in this area, owing to risky fertility behaviors, the cultural taboo against contraception use, and insufficient health infrastructures. HRFBs are also common in the area due to child marriage, rape, and harmful sexual behaviors in elementary school [12–15].

Furthermore, nutritional problems among children under the age of five are common, as evidenced by the magnitude of stunting (36.7%) and anemia (60%) [16, 17]. Maternal HRFBs was one of the major contributors to infant malnutrition. For example, children who were born from women with high-risk fertility behavior had 40 percent and 43 percent more likely to suffer from stunting and anemia, respectively [16, 17]. As a result, a better understanding of the factors linked to risky fertility behavior and its consequences for child malnutrition may aid in the development of interventions. HRFB is more prevalent in low-income countries due to widespread poverty, a lack of basic health services, and early child marriage. Whilst interventions are challenging due to a lack of information about the magnitude and determinants of high-risk fertility behavior in the East Africa region.

Thus, this study aimed to discover factors that influence high-risk fertility behaviors and associations with child stunting and anemia. As maternal and child health is at the top of the region’s agenda, the findings of this study may help to incorporate efforts at the Intergovernmental Authority for Development (IGAD) and African Union level.

## Method

### Data sources

This study was based on the secondary data from nine East African Demography and Health the most recent Survey (Burundi, Ethiopia, Malawi, Mozambique, Rwanda, Tanzania, Uganda, Zimbabwe, and Madagascar) with the analysis period ranged from July 1–30, 2020. The appended datasets of countries were used to estimate the magnitude of high-risk fertility behavior and its effects among reproductive-age women. We included women in this study who had given birth in the five years before the survey and had a child under the age of five.

We used Kids Record (KR) files, which contain information about women and children, for this specific research. In terms of data extraction, we took women who were married and had completed data for the main variables, as well as children’s anthropometric measurements. The data includes socioeconomic, reproductive health, and infant traits such as height for age and hemoglobin level. After data cleaning, the final sample size was 31,873 mothers-children pair who were included in the final analysis. To select study participants in each enumeration region, the DHS used a two-stage stratified sampling technique. We combined data from nine DHS surveys conducted in East African countries, yielding a weighted sample of 31,873 women and children. The strategy is described in detail in the DHS methodology section [16].

### Variables of the study

#### Outcome variables

*Maternal health outcome*. For this study, maternal high-risk fertility behavior was the primary outcome variable which is defined based on several criteria’s as follow;

- High-risk fertility behavior is the outcome of interest for women who gave birth, defined as women age at birth less than 18 or above 34 years or birth interval less than 24 months or high birth order were criteria used to define [16].
- Single high-risk fertility behavior: when a woman reported to had one high-risk fertility behavior the is either younger age less than 18 years, or older age above 34 years, or birth interval less than 24 months, or high-birth order (four and above) [3, 17–19].
- Multiple high-risk fertility behavior: when a woman had a combination of at least two above-mentioned behaviors [3, 17–19]. Unavoidable high-risk fertility behavior is defined as women whose age was between 18 and 34 years and first birth order [16, 17].
- Unavoidable HRFB: when first-order births between ages of 18 and 34 years in women not amenable to the interventions.
- Not in any high-risk category: when women don’t have any risk fertility behavior

*Children health outcomes*. another objective of this study was to see the association between maternal risky fertility behaviors and chronic malnutrition and anemia in children.

- Height-for-age is a measure of linear growth retardation and cumulative growth deficits. Children whose height-for-age Z-score is below minus two standard deviations (-2 SD) from the median of the reference population are considered short for their age (stunted), or chronically undernourished.
- Children who are below minus three standard deviations (-3 SD) are considered severely stunted.
- Anemia is a disease condition marked by low levels of hemoglobin, often below 10g/dl after correction for altitude [17].
- Mildly anemia: when the level of levels of hemoglobin between 10.0 and 10.9 g/dl [17].
- Moderately anemia: when the level of levels of hemoglobin between 7.0 and 9.9 g/dl [17].
- Severe anemia: when the level levels of hemoglobin less than 7g/dl [17].

### Independent variables

Socio-demographic and maternal health services like age group, sex of household headed, women’s educational status, husband’s educational status, maternal occupation status, marital status, media exposure, wealth status, sex of the child, birth order, antenatal care visits, sources of family planning, postnatal care visit, place of delivery, birth attendants, and healthcare access problems were independent variables.

#### Data management and analysis

After extracting the variables based on literature, data from the nine East African countries were combined. To restore the representativeness of the survey and take sampling design into account when calculating standard errors and reliable estimates, the data were weighted using sampling weight, main sampling unit, and strata before any statistical analysis. STATA version 14 was used to perform cross-tabulations and summary statistics.

Using a forest plot, the overall magnitude of high-risk fertility behavior, stunting, and anemia was estimated with the 95 percent Confidence Interval (CI). The DHS data had a hierarchical structure for the determinant factors, which contradicts the classical logistic regression model’s independence of observations and equal variance assumptions. As a result, children are nested within a cluster, and we anticipate that children in the same cluster will be more similar than children across the country. This means that advanced models should be used to account for the variability between clusters. As a result, a mixed effect logistic regression model was fitted (with both fixed and random effects). Standard logistic regression and Generalized Linear Mixed Models (GLMM) were used because the outcome variable was binary (presence or absence of high-risk fertility behavior in women, stunting, and anemia in children). Since the models were nested, the Intra-class Correlation Coefficient (ICC), Likelihood Ratio (LR) test, Median Odds Ratio (MOR), and deviance (-2LLR) values were used to compare and assess model fitness. It was decided to use the model with the lowest deviance. As a result, the mixed-effect logistic regression model fits the data the best. In the multivariable mixed-effect logistic regression model, variables with a p-value of less than 0.2 in the bivariable analysis were considered. The multivariable model used Adjusted Odds Ratios (AOR) with a 95 percent Confidence Interval (CI) and p-value 0.05 to declare major factors high-risk fertility behavior. A multivariable Generalized Linear Mixed Models (GLMM) model was also fitted to see the relationship between HRFB and infant stunting and anemia. The HRFB had a major impact on stunting and anemia, as measured by the AOR with 95 percent confidence intervals and variables with a p-value less than 0.05.

#### Ethical clearance and consent to participate

Measure DHS provided ethical clearance after filling out a request for data access form. The data used in this study is aggregated secondary data that is publicly accessible and does not contain any personal identifying information that can be related to study participants. The data was kept confidential in an anonymous manner.

## Result

### Socio-demographic characteristics

A total of 31,873 study participants were drawn from nine East African countries, with Ethiopia, Tanzania, Madagascar, Burundi, Malawi, and Zimbabwe accounting for 21.8%, 15.6%, 12 percent, 11.3%, 10.9%, and 10.3%, respectively. The median age of respondents was 29 years, with an IQR of 25 to 35, and half of them aged between 25 and 35 years. The majority (80.5%) of women came from rural areas, nearly one-third (32.2%) had no formal schooling, and 45.4 percent lived in poverty (Table 1).

**Table 1:** Socio-demographic characteristics of reproductive age women in east Africa region.

Characteristics	Frequency	Percentage
**Country**		
Burundi	3,631	11.4
Ethiopia	6,935	21.8
Malawi	3,492	11
Mozambique	2,254	7.1
Rwanda	1,701	5.3
Tanzania	4,976	15.6
Uganda	1,760	5.5
Zimbabwe	3,313	10.4
Madagascar	3,811	12
**Age of respondents**		
15–19	1,168	3.7
20–24	6,413	20.1
25–29	8,782	27.6
30–34	7,341	23
35–39	5,044	15.8
40–44	2,393	7.5
45–49	732	2.3
**Residence**		
Urban	6,899	19.3
Rural	28,785	80.7
**Women level of education**		
No formal education	10259	32.2
Primary school	14893	46.7
Secondary school	5930	18.6
Diploma and higher	791	2.5
**Husband education**		
No formal education	8034	25.2
Primary school	15239	47.8
Secondary school	6977	21.9
Diploma and higher	1623	5.1
**Wealth index**		
Poor	14465	45.4
Middle	5887	18.5
Rich	11521	36.1
**Household head**		
Male	26892	84.4
Female	4981	15.6
**Media exposure**		
Yes	19653	60.3
No	12653	39.7
Health insurance coverage		
Yes	2309	7.6
No	27863	92.4
Women working condition		
Yes	22055	69.2
No	9818	30.8
Husband working condition		
Yes	30073	94.4
No	1800	5.6

#### Reproductive history of women

The majority of the participants (88.4%) were multiparous, almost two-thirds (65.6%) gave birth in the health facilities, and about 4.5 percent gave birth by cesarean section. The majority (62.2%) had ANC follow-up, 21.2% of women had also postnatal follow-up, 40.3% of women had family planning details from the media, and 35.4% had discontinued family planning in the five years preceding the survey. More than two-thirds (68.2%) of women had trouble accessing healthcare due to a lack of resources, distance, permission, or companionship (Table 2).

**Table 2:** Reproductive characteristics of child bearing women in East Africa region.

Characteristics	Frequency	Percentage
**Parity**		
Primiparous	3699	11.6
Multiparous	28174	88.4
**Age at first birth**		
Less than 18	1553	14.9
18–34 years	25314	79.4
Above 34	5006	15.7
**Place of delivery**		
Home	10877	34.1
Health facility	20996	65.9
**History of abortion**		
Yes	4336	13.6
No	27537	86.4
**Current contraceptive use**		
Yes	18434	57.8
No	13439	42.2
**The average size at birth**		
Small	5505	17.3
Average	15962	50.1
Large	10403	32.6
**Delivered Cesarean section**		
Yes	1432	4.5
No	30391	95.5
A faced healthcare access problem		
Yes	21748	68.2
No	10125	31.8
ANC follow up		
Yes	19825	62.2
No	12048	37.8
Postnatal follow-up		
Yes	6743	21.2
No	25130	78.8
Sex of child		
Male	15980	50.1
Female	15893	49.9
Discontinued contraceptive methods		
Yes	11,283	35.4
No	20,590	64.6
Know the source of family planning		
Yes	14,184	55.5
No	17,689	44.5
Had information about family planning		
Yes	12,853	40.3
No	19,020	59.7

### High-risk fertility behavior

**Fig 1:**
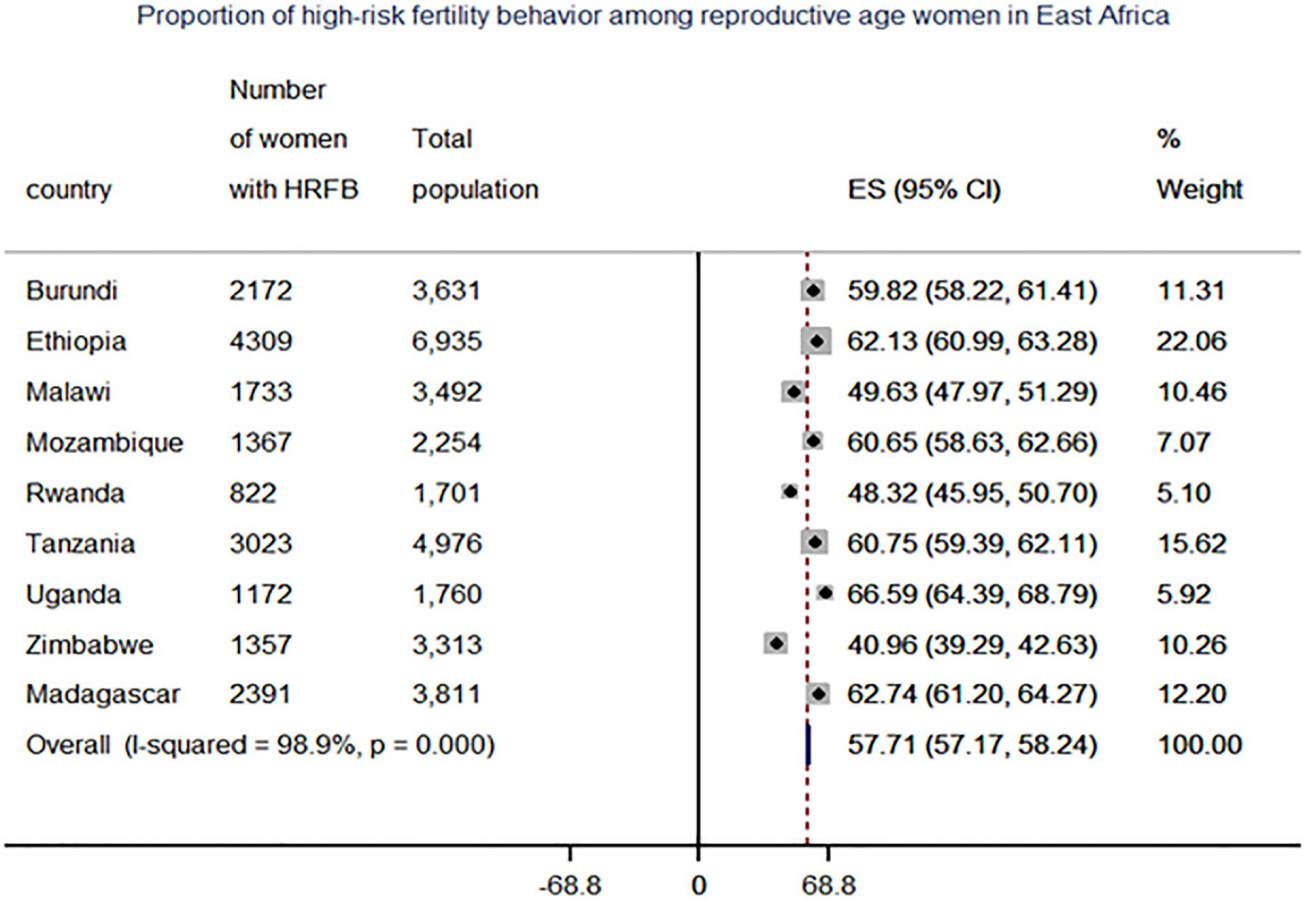
Forest plot of proportion of high-risk fertility behavior among reproductive-age women in East Africa countries.

**Fig 2:**
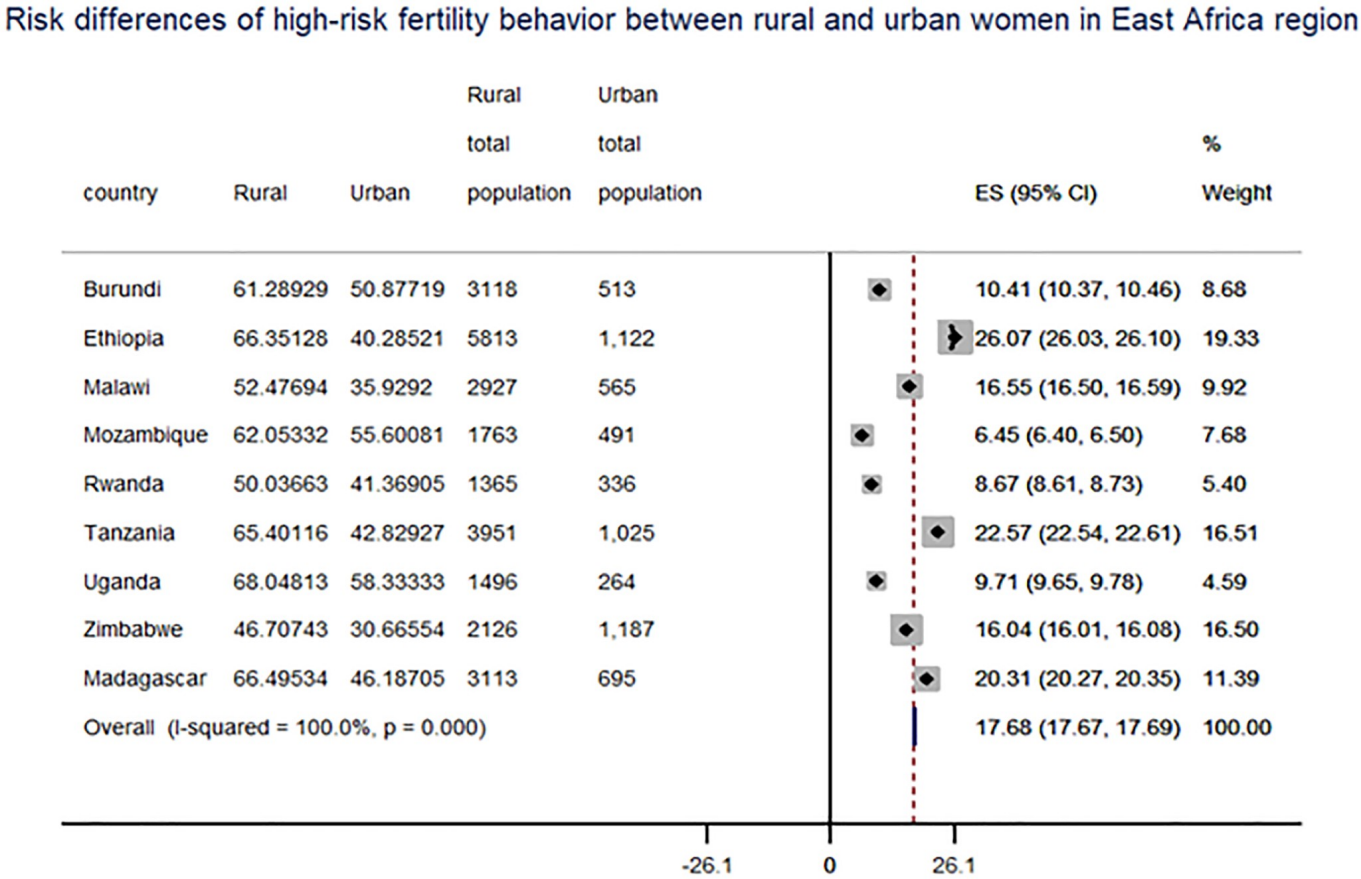
Forest plot of risk differences of high-risk fertility behavior among between rural and urban reproductive-age women in East Africa countries.

The pooled analysis of this study indicated that 57.6% (95 percent CI: 57.7 to 58.2) of women had at least one high-risk fertility behavior, while 21.6 percent had multiple risk factors. The most common single high-risk fertility activity was higher birth order (45%), older age at birth (over 34 years) (15.7%), and birth period shorter than 24 months (15.6%). A combination of older women’s age and higher birth order (age over 34 and birth order above 3) and a birth period less than 24 months and birth order above 3 accounted for 14.5 percent and 8.7% of women, respectively. Within the country, there was also variance, ranging from 66.59% in Uganda to 41% in Zimbabwe (Fig 1). Significant variations were also found between women from rural and urban areas, with risk differences of 17.68% and 7.21% for single and multiple high-risk fertility behaviors in the East Africa region, respectively (Fig 2) and (Table 3).

**Table 3:** High-risk fertility behavior of reproductive age women in east Africa region.

Characteristics	Frequency	Percentage
**Any high-risk behavior**		
Yes	18346	57.6
No	13527	42.4
**Single high-risk fertility behavior**		
Age less than 18 years	1553	4.9
Age above 34 years	5006	15.7
Birth order above 3	14351	45
The birth interval of less than 24 months	4963	15.6
**Multiple high-risk fertility behavior**		
Age less than 18 years and birth interval less than 24 months	121	0.4
Age above 34 years and birth interval less than 24 months	662	2.1
Age above 34 years and birth order above 3	4619	14.5
Birth interval less than 24 months and birth order above 3	2768	8.7
Age above 34 years and birth interval less than 24 months, and birth order above 3	643	2
**Unavoidable risk category**		
First birth order and age of mother between 18 and 34 years	4630	14.5
**No high-risk fertility behavior**	10,928	34.3

### Factors associated with high-risk fertility behavior

In the multivariable mixed-effect logistic regression model, mother and husband education levels, residence, country, wealth status, sex of household, place of delivery, delivered by CS, abortion, healthcare access problems, currently contraceptive utilization were variables correlated with high-risk fertility behaviors at a p-value of 0.05. In contrast to uneducated mothers, the chances of high-risk pregnancy activity were reduced by 41% (AOR = 0.59, 95% CI: 0.56 to 0.64), 68 percent (AOR = 0.32, 95% CI: 0.29 to 0.36), and 76% (AOR = 0.24, 95% CI: 0.19 to 0.29) for women who completed primary, secondary, and certificate and higher-level schooling. For those husbands who attended primary, secondary, diploma, and above level of education, the odds of high-risk fertility behaviors were reduced by 11% (AOR = 0.89, 95% CI: 0.83 to 0.95), 29% (AOR = 0.71, 95% CI: 0.65 to 0.78), and 25% (AOR: 0.75, 95% CI: 0.65 to 0.87) compared to low level of education, respectively.

Female-headed households had an 11% lower risk of high-risk fertility activity than male-headed households (AOR = 0.89, 95% CI: 0.83 to 0.95). Furthermore, rural mothers had a 1.26-fold higher probability of fertility activity than city mothers (AOR = 1.26, 95% CI: 1.17 to 1.36). Similarly, the chances of high-risk fertility activity were 1.10 times higher in wealthy women than in poor women (AOR = 1.10, 95%CI: 1.03 to 1.18). In addition, women with healthcare access challenges had a 10% higher risk of high-risk fertility activity than mothers who did not have such a history (AOR = 1.14, 95% CI: 1.08 to 1.20). Women who had terminated pregnancies had a 16 percent increased risk of high-risk fertility activity relative to those who had no such history (AOR = 1.16, 95% CI: 1.08 to 1.25). The chances of high-risk fertility activity were 1.51 times higher for mothers who gave birth at home (AOR = 1.51, 95% CI: 1.41 to1.61) than for mothers who gave birth in a hospital (AOR = 1.51, 95% CI: 1.41 to1.61). Furthermore, women who had antenatal care follow-up with their recent baby had a 16% higher risk of delivering a healthy baby than those who did not (AOR = 1.16, 95% CI: 1.10 to 1.23). In contrast, mothers who were aware of the sources of family planning had an 11% lower risk of high-risk fertility activity than those who were not aware of the sources of family planning (AOR = 0.89, 95% CI: 0.79 0.97). Women who gave birth by cesarean section had a 30% lower risk of high-risk fertility activity than women who gave birth vaginally (AOR = 0.70, 95% CI: 0.63 to 0.79). Similarly, women who were currently using contraception were reduced by 10% compared to those who were not currently using contraception (AOR = 0.90, 95% CI: 0.85 to 0.95). (Table 4).

**Table 4:** Factors associated with high-risk fertility behavior among women gave birth in east Africa region.

Characteristics	Odds ratio		Characteristics	Crude 95%CI	Adjusted 95%CI
	Crude 95%CI	Adjusted 95%CI	Current contraceptive utilization		
**Country**			Yes	0.62(0.59 0.65)	0.90(0.85 0.95)*
Burundi	2.14(1.94 2.36)	0.93(0.83 1.06)	No	1	1
Ethiopia	2.32(2.13 2.53)	0.73(0.65 0.83)*	**Sex of household**		
Malawi	1.40(1.27 1.55)	0.87(0.78 0.97)*	Male	1	1
Mozambique	2.23(2.0 2.49)	1.06(0.92 1.21)	Female	0.77(0.72 0.82)	0.89(0.83 0.95)*
Rwanda	1.35(1.20 1.52)	0.77(0.67 0.88)*	**Faced healthcare access problem**		
Tanzania	2.20(2.01 2.41)	1.09(0.98 1.22)	**Yes**	1.56(1.48 1.63)	1.14(1.08 1.20)*
Uganda	2.85(2.52 3.23)	1.68(1.46 1.93)*	**No**	1	1
Madagascar	2.42(2.19 2.67)	1.06(0.94 1.19)	**Delivered by CS**		
Zimbabwe	1	1	**Yes**	0.42(0.38 0.48)	0.70(0.63 0.79)*
**Women level of education**			**No**	1	1
No formal education	1	1	**Residence**		
Primary	0.54(0.51 0.57)	0.59(0.56 0.64)*	**Urban**	1	1
Secondary	0.23(0.22 0.25)	0.32(0.29 0.36)*	**Rural**	2.20(2.07 2.33)	1.26(1.17 1.36)*
Higher	0.13(0.11 0.16)	0.24(0.19 0.29)*	**Media exposure**		
**Husband level of education**			**Yes**	0.65(0.62 0.68)	0.97(0.92 1.03)
No formal education	1	1	**No**	1	1
Primary	0.68(0.64 0.72)	0.89(0.83 0.95)*	**Postnatal care follow up**		
Secondary	0.34(0.32 0.36)	0.71(0.65 0.78)*	**Yes**	0.71(0.67 0.75)	0.98(0.92 1.05)
Higher	0.24(0.22 0.27)	0.75(0.65 0.87)*	**No**	1	1
**Wealth status**			**ANC follow up**		
Poor	1	1	**Yes**	0.84(0.81 0.88)	1.16(1.10 1.23)*
Middle	0.86(0.80 0.91)	1.03(0.96 1.10)	**No**	1	1
Rich	0.54(0.51 0.57)	1.10(1.03 1.18)*	**Know sources of family planning**		
**Ever terminated pregnancy**			**Yes**	0.71(0.68 0.74)	0.89(0.79 0.97)*
Yes	1.14(1.07 1.22)	1.16(1.08 1.25)*	**No**	1	1
No	1	1			
**Place of delivery**					
Home	2.04(1.94 2.14)	1.51(1.41 1.61)*			
Health facility	1	1			

#### Association between maternal high-risk fertility behaviors and stunting and anemia in children

To investigate the relationship between high-risk fertility activity and infant stunting and anemia, a mixed effect generalized linear mixed model (GLLM) was fitted. Thus, mothers under the age of 18 and over the age of 34, birth order greater than three, birth interval, and interactions of higher birth order and age greater than 34 years are associated with anemia stunting. Stunting was 1.55 (AOR = 1.55, 95% CI: 1.39 to 1.73), 1.33 (AOR = 1.33, 95% CI: 1.21 to 1.46) and 1.25 (AOR = 1.25, 95% CI: 1.18 to 1.32) times more likely in children born to mothers under the age of 18 at the time of birth, birth period less than 24 months, and birth order above three. Similarly, Similarly, an interactions of mother age over 34 and birth order greater than 3 was related to a 1.35 higher risk of infant stunting than those that did not have these characteristics (AOR = 1.35, 95 percent CI: 1.06 to 1.73). On other hand a mother’s age at birth for 34 years is associated with a 25% lower risk of child stunting (AOR = 0.75, 95 percent CI: 0.60 to 0.95), compared to other age groups.

When the mother was less than 18 years old at the time of birth, the birth span was less than 24 months, and the birth order was greater than 3, the odds of infant anemia were 1.19 (AOR = 1.19, 95 percent CI: 1.07 to 1.33), 1.12 (AOR = 1.12, 95 percent CI: 1.01 to 1.23), and 1.26 times higher than their counterparts. Women over 34 years old had a 28 percent lower risk of infant anemia than women of other ages (AOR = 0.72, 95 percent CI: 0.58 to 0.90) (Table 5).

**Table 5:** Effect of high-risk fertility behavior on child chronic malnutrition and Anemia.

High-risk fertility behaviors	Stunting		Crude OR	Adjusted OR	Anemia		Crude OR	Adjusted OR
	Yes	No			Yes	No		
Age less than 18 years								
Yes	711	842	1.35(1.21 1.50)	1.55(1.39 1.73)*	877	676	1.08(0.98 1.20)	1.19(1.07 1.33)*
No	18633	11687	1	1	16391	13929	1	1
Age above 34 years								
Yes	2022	2984	1.07(1.01 1.14)	0.75(0.60 0.95)*	2660	2346	0.95(0.90 1.20)	0.72(0.58 0.90)*
No	10376	16491	1	1	14608	12259	1	1
Birth interval less than 24 months								
Yes	2160	2803	1.26(1.18 1.34)	1.33(1.21 1.46)*	8161	2103	1.15(1.08 1.23)	1.12(1.01 1.23)*
No	10238	16672	1	1	9107	12502	1	1
Birth order 4 and above								
Yes	5950	8401	1.21(1.16 1.27)	1.25(1.18 1.32)*	2860	6190	1.21(1.16 1.26)	1.26(1.19 1.34)*
No	6448	11074	1	1	14408	8415	1	1
Age >34 years and Birth order >3								
Yes	1907	2712	1.12(1.05 1.19)	1.35(1.06 1.73)*	2493	2126	0.99(0.93 1.06)	1.19(0.95 1.50)
No	10491	16763	1	1	14775	12479	1	1
Age < 18 years & birth interval <24 months								
Yes	58	63	1.44(1.00 2.07)	0.81(0.55 1.20)	72	49	1.24(0.86 1.80)	1.03(0.69 1.52)
No	12340	19412	1	1	17196	14556	1	1
Age >34 years & birth interval <24 months								
Yes	278	384	1.15(0.98 1.35)	1.43(0.55 3.73)	363	299	1.02(0.87 1.19)	1.03(0.40 2.65)
No	19091	12120	1	1	16905	14306	1	1
Birth interval <24 months and birth order >3								
Yes	1233	1535	1.30(1.20 1.41)	0.92 (0.80 1.05)	1658	1110	1.26(1.16 1.37)	1.03(0.90 1.18)
No	11165	17940	1	1	15610	13495	1	1
Age above 34, birth order >3, and birth interval <24 months								
Yes	270	373	1.15(0.98 1.35)	0.59(0.22 1.59)	354	289	1.03(0.87 1.20)	0.86(0.33 2.26)
No	12128	19102	1	1	16914	14316	1	1

## Discussion

This study intended to determine the pooled estimates of high-risk fertility behavior in East Africa countries. Thus, the pooled analysis revealed that 57.7% and 21.6% of women who gave birth had at least one and multiple high-risk fertility behavior. Of which, higher birth order, age above 34 at birth, and birth interval less than 24 months were the common single high-risk fertility behaviors. Moreover, significant variations were also observed among countries ranged from 41% in Zimbabwe to 66% in Uganda. Likewise, a significant difference was also observed between rural and urban mothers in terms of high-risk fertility behavior which accounted for 17% of risk differences (RD). The possible explanations for the observed variation might be child marriage practices, a high magnitude of unmet need for family planning, and bad cultural myths and beliefs to use family planning among women. In addition, most of the countries in Africa had no demography and population policy despite rapid population growth. In addition, these findings suggest that more interventions which focus on maternal health services like provision of family planning and counseling on reduction of risky fertility behaviors are very important.

Furthermore, there were also substantial risk variations in high-risk fertility activity between rural and urban areas. This may be explained by a lack of access to healthcare and family planning, suggesting that rural areas are the best place to participate to minimize maternal mortality, meet sustainable development goals, and achieve universal health coverage. This result was in line with results from Nepalese and Indian studies [20–22]. Women and husbands with some degree of schooling had lower risky-fertility behavior than women with no formal education, according to this report. This result was in line with the findings of other studies [20, 21, 23]. Women’s awareness about the benefits of birth spacing and reproductive health attributes expanded as their educational levels rose. The effects of school reproductive clubs and the inclusion of fertility biology in the educational curriculum may also explain this. In contrast to male-headed households, female-headed households have a lower risk of high-risk fertility activity. This may be because women are responsible for both earning a living and caring for their children

Rich women, on the other hand, are more likely than poor women to participate in high-risk fertility activity. This may be because wealthier women (households) could want more children, which could contribute to risky fertility activity. This result was in line with previous research. Significant regional differences in high-risk fertility behavior were also discovered in this research. Women from Uganda had 1.68 times more likely to had high-risk fertility behavior than women from Zimbabwe, while women from Rwanda, Malawi, and Ethiopia had 23%, 13%, and 27% lower chances of high-risk fertility behavior than Zimbabwe, respectively. Regarding the place of delivery, women who gave birth at home had a greater high-risk fertility behavior than women who gave birth at a health facility [6, 7, 24, 25]. This result was in line with those of previous Ethiopian studies [26]. Immediate post-partum family planning programs, such as ICUD, were often available via health facilities. Integration and strengthening of family planning services with obstetrics services like IUCD insertion immediately after delivery.

Similarly, women who had ever terminated a pregnancy (abortion history) were more likely to engage in high-risk fertility activity than those who had not. This result was in line with previous results in Sub-Saharan Africa [9, 27]. Abortion was commonly associated with unwanted pregnancies with shorter birth periods and pregnancies at a young age, and it represented a lack of contraception use, which affected high-risk fertility. Women who had trouble accessing health services were often more likely to participate in high-risk fertility behaviors. This finding was consistent with previous research [28]. This may be because women who had trouble accessing health services used less family planning and received less ANC and postnatal care, resulting in shorter birth periods, births at an older age, and high birth orders. In addition, this study showed that mothers who received ANC during pregnancy had an increased risk of high-risk fertility activities relative to those who did not. This result contradicted previous research. This may be because women with shorter birth periods and conceptions at a later age are frequently high-risk and need regular monitoring and follow-up.

Another result of this study was that cesarean section delivery is associated with lower risky fertility activity as compared to vaginal delivery. This may be because frequent Cesarean section deliveries reduced the number of pregnancies due to the possibility of negative effects of repeated CS [29]. Similarly, during data collection, women who understood the origins of modern contraception and existing users were associated with lower risk fertility activity. This result was consistent with previous research [3, 5]. A strong understanding of contraceptive strategies and their use decreased unintended pregnancy and improved birth intervals.

This research, on the other hand, discovered a correlation between high-risk fertility activity and childhood chronic stunting and anemia. Thus, in the East African region, the prevalence of stunting and anemia was 38.9 percent and 54.2 percent, respectively, among those who gave birth in the five years preceding the study. In this report, the prevalence of chronic malnutrition (stunting) was lower than in India (45.1 percent) and Nepal (39.7 percent) [30]. This finding, however, was higher than that of Bangladesh (36%) and three disadvantaged east African countries (36.7%) [19, 31]. Furthermore, the incidence of infant anemia was lower than a study finding of 43.7 percent of Bangladeshis. This finding reflects that maternal fertility behaviors are also contributors to nutritional problems among children. Socio-cultural disparities, such as cultural taboos against some food products in Southeast Asia, maybe one reason. Another point to remember is the correlation between high-risk fertility and chronic malnutrition in children. As a result, women under the age of 18 at the time of birth were related to a higher risk of stunting and anemia. This result was in line with previous research. Birth age is often linked to social and health disadvantages and inequality. In comparison to other age classes, women over 34 years old at the time of birth have a lower risk of stunting and anemia.

Furthermore, children with a birth order greater than 3 and a birth interval of less than 24 months have a higher risk of stunting and anemia. This result was in line with previous research. This may be attributed to a short birth period and a high birth order, which is related to intrauterine growth retardation in infants, maternal anemia, and maternal stress, both of which contribute to prematurity. Similarly, women with multiple high-risk fertility behaviors, such as age over 34 and high birth order of three or more, had a higher occurrence of high-risk fertility activity than those who did not. This finding was consistent with previous research. In general, this study found that high-risk fertility activity is widespread among East African reproductive-age women. Material high-risk fertility activity is linked to chronic malnutrition and anemia in children. This indicates that growing contraceptive use by women of childbearing age would help both the mother and the child’s health.

For evidence-based approaches, this research has implications for reproductive-age women, healthcare planners, and policymakers. Furthermore, the results of this study revealed that amenable variables such as home delivery, educational status, wealth status, and contraceptive usage could be the target area for resolving the issues. In addition, factors such as schooling, residency, and family planning source have been described as strategies for reducing maternal and child mortality. However, there are some drawbacks to this research. First, the study’s cross-sectional nature influenced the cause-effect relationship; second, health system characteristics were not assessed; and finally, the data in this study had recall bias issues, such as the number of months between births.

## Conclusion

This study revealed that the magnitude of high-risk fertility behavior was higher in the region. The finding of this study underscores that interventions focused on health education and behavioral change of women, and improvement of maternal healthcare access would be helpful to avert risky fertility behaviors. In brief, encouraging contraceptive utilization and creating awareness about birth spacing among reproductive-age women would be more helpful. Meanwhile, frequent nutritional screening and early intervention of children born from women who had high-risk fertility characteristics are mandatory to reduce the burden of chronic malnutrition.

## Abbreviations

ANC: Antenatal Care
AOR: Adjusted Odds Ratio
CI: Confidence Interval
CS: Cesarean Section
DHS: Demographic and Health Survey
EA: Enumeration Area
E: East
DHS: Ethiopian Demographic and Health Survey
ICC: Intra Class Correlation
IUD: Intrauterine Device
KR: Kids Record
LLR: Likelihood Ratio
MOR: Median Odds Ratio
N: North
PCV: Proportion of Cluster Variance
SE: Standard Error
SGD: Sustainable Development Goal
SSA: Sub-Saharan Africa

## References

[1] Ogundari K. and Awokuse T., Human capital contribution to economic growth in Sub-Saharan Africa: does health status matter more than education? Economic Analysis and Policy, 2018. 58: p. 131–140.

[2] Bongaarts J., Development: Slow down population growth. Nature, 2016. 530(7591): p. 409–412. doi: 10.1038/530409a 26911766

[3] Hammarberg K., et al., Fertility-related knowledge and information-seeking behaviour among people of reproductive age: a qualitative study. Human Fertility, 2017. 20(2): p. 88–95. doi: 10.1080/14647273.2016.1245447 27778517

[4] Isabirye A., The effects of high risk fertility behavior on child survival in Uganda. 2012, Makerere University.

[5] Khan T. and Ali Khan R.E., Fertility behaviour of women and their household characteristics: A case study of Punjab, Pakistan. Journal of human ecology, 2010. 30(1): p. 11–17.

[6] Balasch J. and Gratacós E., Delayed childbearing: effects on fertility and the outcome of pregnancy. Current Opinion in Obstetrics and Gynecology, 2012. 24(3): p. 187–193. doi: 10.1097/GCO.0b013e3283517908 22450043

[7] Brown W., et al. Impact of family planning programs in reducing high-risk births due to younger and older maternal age, short birth intervals, and high parity. in Seminars in perinatology. 2015. Elsevier. 10.1053/j.semperi.2015.06.006 26169538

[8] Casterline J.B. and Lazarus R., Determinants and consequences of high fertility: a synopsis of the evidence. Addressing the Neglected MDG: World Bank Review of Population and High Fertility, World Bank publications, 2010.

[9] Dibaba Y., Child spacing and fertility planning behavior among women in mana district, Jimma Zone, South West Ethiopia. Ethiopian journal of health sciences, 2010. 20(2). doi: 10.4314/ejhs.v20i2.69433 PMC3275838 22434965

[10] Fall C.H., et al., Association between maternal age at childbirth and child and adult outcomes in the offspring: a prospective study in five low-income and middle-income countries (COHORTS collaboration). The Lancet Global Health, 2015. 3(7): p. e366–e377. doi: 10.1016/S2214-109X(15)00038-8 25999096 PMC4547329

[11] Rutstein S.O. and Winter R., Contraception needed to avoid high-fertility-risk births, and maternal and child deaths that would be averted. 2015: ICF International.

[12] Rutstein S.O. and Winter R., The effects of fertility behavior on child survival and child nutritional status: Evidence from the Demographic and Health Surveys 2006 to 2012. 2014.

[13] Jonas K., et al., Teenage pregnancy rates and associations with other health risk behaviours: a three-wave cross-sectional study among South African school-going adolescents. Reproductive health, 2016. 13(1): p. 50. doi: 10.1186/s12978-016-0170-8 27142105 PMC4855358

[14] Marshall E., et al., Child marriage in Ethiopia: A review of the evidence and an analysis of the prevalence of child marriage in hotspot districts. 2016, UNICEF Ethiopia and Overseas Development Institute (ODI).

[15] Way, C., The millennium development goals report 2015. 2015: UN.

[16] Program, D., DHS Methodology. 2017.

[17] Central Statistical Agency, Ethiopia demographic and health survey 2016, in ORC Macro, Calverton, Maryland, USA. 2016.

[18] Dejene E.G.a.T., Correlates of High Risk Fertility Behaviour in Ethiopia: A Multilevel Analysis of the 2011 Ethiopian Demographic and Health Survey Data. Journal of Health, Medicine and Nursing, 2017. 39(ISSN 2422-8419).

[19] Rahman M., et al., Maternal high-risk fertility behavior and association with chronic undernutrition among children under age 5 y in India, Bangladesh, and Nepal: Do poor children have a higher risk? Nutrition, 2018. 49: p. 32–40. doi: 10.1016/j.nut.2017.10.001 29735148

[20] Adhikari R., Demographic, socio-economic, and cultural factors affecting fertility differentials in Nepal. BMC pregnancy and childbirth, 2010. 10(1): p. 19. 20426863 10.1186/1471-2393-10-19 PMC2885993

[21] Asghar M., Murry B., and Saraswathy K.N., Fertility behaviour and effect of son preference among the Muslims of Manipur, India. Journal of Anthropology, 2014. 2014.

[22] Kulu H., Why do fertility levels vary between urban and rural areas? Regional studies, 2013. 47(6): p. 895–912.

[23] Hammarberg K., et al., Men’s knowledge, attitudes and behaviours relating to fertility. Human Reproduction Update, 2017. 23(4): p. 458–480. doi: 10.1093/humupd/dmx005 28333354

[24] Adiri F., et al., Fertility behaviour of men and women in three communities in Kaduna state, Nigeria. African Journal of Reproductive Health, 2010. 14(3): p. 97–105.

[25] Hasan A., Singh M.K., and Khan A., Fertility behaviour and contraceptive use in urban slums of district Gorakhpur. International Journal Of Community Medicine And Public Health, 2017. 4(12): p. 4702–4705.

[26] Alemu T., Assessment of Relationship between Infant Death and High Risk Fertility Behavior among Married Women of Afar. Zone Four. 2006, Addis Ababa University.

[27] Stover J. and Ross J., Changes in the distribution of high-risk births associated with changes in contraceptive prevalence. BMC Public Health, 2013. 13(S3): p. S4. doi: 10.1186/1471-2458-13-S3-S4 24564577 PMC3847521

[28] Ralph L.J. and Brindis C.D., Access to reproductive healthcare for adolescents: establishing healthy behaviors at a critical juncture in the lifecourse. Current opinion in obstetrics and gynecology, 2010. 22(5): p. 369–374. doi: 10.1097/GCO.0b013e32833d9661 20733485

[29] Bayrampour H. and Heaman M., Advanced maternal age and the risk of cesarean birth: a systematic review. Birth, 2010. 37(3): p. 219–226. doi: 10.1111/j.1523-536X.2010.00409.x 20887538

[30] Rahman M., Hosen A., and Khan M.A., Association between Maternal High-Risk Fertility Behavior and Childhood Morbidity in Bangladesh: A Nationally Representative Cross-Sectional Survey. The American journal of tropical medicine and hygiene, 2019: p. tpmd190221. doi: 10.4269/ajtmh.19-0221 31333165 PMC6779183

[31] Raj A., et al., The effect of maternal child marriage on morbidity and mortality of children under 5 in India: cross sectional study of a nationally representative sample. Bmj, 2010. 340: p. b4258. doi: 10.1136/bmj.b4258 20093277 PMC2809839

