# Supplementary material for: Determinants of maternal high-risk fertility behaviors and its correlation with child stunting and anemia in the East Africa region: A pooled analysis of nine East African countries
Source: PLoS One. 2021 Jun 30;16(6):e0253736. doi: 10.1371/journal.pone.0253736 (PMC8244896; doi:10.1371/journal.pone.0253736)
Supplement: Attachment — Submitted filename: Point by point response.docx [file pone.0253736.s001.docx]

**Point by point response**

**Manuscript title: Determinants of maternal high-risk fertility behaviors and its correlation with child stunting and anemia in the East Africa region: A Pooled Analysis of nine East African countries**

**Manuscript ID:** PONE-D-20-27151

**Journal – PLOS ONE**

**Dear editor/reviewer**

Dear all,

We would like to thank you for this constructive, building, and improvable comments on this manuscript that would improve the substance and content of the paper. We considered each comment and clarification questions of editors and reviewers on the document thoroughly. Our point-by-point responses for each comment and issues are described in detail on the following pages. Further, the details of changes were shown by track changes in the supplementary document attached.

Koku Sisay Tamirat

On behalf of all authors

**Editor comments**

When submitting your revision, we need you to address these additional requirements.

1. Please ensure that your manuscript meets PLOS ONE's style requirements, including those for file naming. The PLOS ONE style templates can be found at https://journals.plos.org/plosone/s/file?id=wjVg/PLOSOne_formatting_sample_main_body.pdf and<https://journals.plos.org/plosone/s/file?id=ba62/PLOSOne_formatting_sample_title_authors_affiliations.pdf>

- Author response:  Thanks editors for your constructive comments, based on your comments we made all corrections according to Submission guidelines.

2. Please include in your Methods section the date ranges of the DHS database analysed in the current study.

- Author response:  Thanks editor for your comments for the purpose of this study we used secondary data sources from measure Demography and Health Survey (DHS) website with after fill the request form. The date of analysis for this study was from July 1-30, 2020. Mentioned in the method section, page 4, Line 88-89.

We note that you have indicated that data from this study are available upon request. PLOS only allows data to be available upon request if there are legal or ethical restrictions on sharing data publicly. For information on unacceptable data access restrictions, please see http://journals.plos.org/plosone/s/data-availability#loc-unacceptable-data-access-restrictions.

- Author response: Thanks reviewer for your constructive comments which are highly important to improve the quality of manuscript quality.

In your revised cover letter, please address the following prompts:

a) If there are ethical or legal restrictions on sharing a de-identified data set, please explain them in detail (e.g., data contain potentially identifying or sensitive patient information) and who has imposed them (e.g., an ethics committee). Please also provide contact information for a data access committee, ethics committee, or other institutional body to which data requests may be sent.

- Author response:  Thank you editor for your constructive comments. This study is further analysis of publicly available secondary data sources from measure DHS. Ethical clearance was obtained after filling an online data acquisition form of measure DHS. The request form available at [www.measuredhs.com](http://www.measuredhs.com).

b) If there are no restrictions, please upload the minimal anonymized data set necessary to replicate your study findings as either Supporting Information files or to a stable, public repository and provide us with the relevant URLs, DOIs, or accession numbers. Please see http://www.bmj.com/content/340/bmj.c181.long for guidelines on how to de-identify and prepare clinical data for publication. For a list of acceptable repositories, please see http://journals.plos.org/plosone/s/data-availability#loc-recommended-repositories.

- Author response: Corrected in the main document of the manuscript. Mentioned in the declaration section of the manuscript, page 17, line 357-358.

We will update your Data Availability statement on your behalf to reflect the information you provide.

 We note you have included a table to which you do not refer in the text of your manuscript. Please ensure that you refer to Table 3 in your text; if accepted, production will need this reference to link the reader to the Table.

- Author response: Corrected in the main document. Thank you editors for your insightful comments. it is already mentioned in the main document, page 9, line 188-189.
  Reviewer 1: Is the manuscript technically sound, and do the data support the conclusions?
  The manuscript must describe a technically sound piece of scientific research with data that supports the conclusions. Experiments must have been conducted rigorously, with appropriate controls, replication, and sample sizes. The conclusions must be drawn appropriately based on the data presented.

Reviewer #1: Partly

- Author response: The conclusion section corrected based on the results and objectives of the study.

Reviewer comments: Is the manuscript presented in an intelligible fashion and written in standard English?
PLOS ONE does not copyedit accepted manuscripts, so the language in submitted articles must be clear, correct, and unambiguous. Any typographical or grammatical errors should be corrected at revision, so please note any specific errors here.

 Reviewer #1: No

- Author response: The language proofread by all authors and other language exerts. Language errors edited and corrected in the main document of the manuscript.

Reviewer comments: Define abbreviations at first mention (e.g HRFB).

- Author response: Corrected in the main document of the manuscript HRFB stands to High-risk fertility behavior among reproductive age women. The detail mentioned in the introduction section and variables of the study section. Mentioned in the Page 3, line 63-64 and page 5, 103-113.

2. In the abstract, what do you mean by "Women and husband education"? please define the direction. Are you saying poor/low women and husband education?

- Author response: Corrected as “*In contrast to uneducated mothers, the chances of high-risk pregnancy activity were reduced by 41 % (AOR=0.59, 95 % CI: 0.56 to 0.64), 68 percent (AOR=0.32, 95 % CI: 0.29 to 0.36), and 76 % (AOR= 0.24, 95 % CI: 0.19 to 0.29) for women who completed primary, secondary, and certificate and higher level schooling. Those husband who attended primary, secondary, diploma and above level of education, the odds of high-risk fertility behaviors were reduced by 11 % (AOR=0.89, 95 % CI: 0.83 to 0.95), 29 % (AOR=0.71, 95 % CI: 0.65 to 0.78), and 25 % (AOR: 0.75, 95 % CI: 0.65 to 0.87) compared to low level of education, respectively.* Corrected in the main document of the manuscript, page 9-10, Line 195-202.

Reviewer’s comments: The abstract's conclusion is not properly written and hard to follow. Please do not repeat the study result in the conclusion section.

- Author response: Thank you reviewer for your constructive comments. The abstract section revised and rephrased. Written as “**Background:** Low contraceptive utilization, child marriage, and a poor health system contributed to a high-risk fertility behavior in the East African region. As a result, this study aimed to establish determinants of high-risk fertility activity and their effect on child stunting and anemia. **Method:** This study relied on secondary data sources from recent demography and health surveys of nine east African countries. Relevant data were extracted from Kids Record (KR) files and appended for the final analysis; 31,873 mother-child pairs were included in the final analysis. The mixed-effect logistic regression model (fixed and random effects) was used to describe the determinants of high-risk fertility behavior (HRFB) and its correlation with child stunting and anemia. **Result:** According to the pooled study, 57.6% (95 % CI: 57.7 to 58.2) of women had at least one high-risk fertility behavior, with major a disparities found across countries and women's residences. High-risk fertility behaviors were more common among women of rural dwellers, faced healthcare access problems, history of abortion, better economic conditions, and had antenatal care follow-up. Consequently, younger women at first birth, narrow birth intervals, and high birth orders were HRFBs associated with an increased occurrence of child stunting and anemia. **Conclusion:** This study revealed that the magnitude of high-risk fertility behavior was higher in the region. The finding of this study underscores that interventions focused on health education and behavioral change of women, and improvement of maternal healthcare access would be helpful to avert risky fertility behaviors. In brief, encouraging contraceptive utilization and creating awareness about birth spacing among reproductive-age women would be more helpful. Meanwhile, frequent nutritional screening and early intervention of children born from women who had high risk fertility characteristics are mandatory to reduce the burden of chronic malnutrition.

4. The following statement in the methods section is confusing: "Whereas to see the relationship of risky behaviors with child chronic malnutrition and anemia, child hemoglobin level, and height for age measurements as dependent variables."
Which are/is the dependent variable(s)? High risk fertility behaviour? or chronic malnutrition? or anemia? or child hemoglobin level?
How were they defined? How were they expressed in the analysis? What is the diference between chronic malnutrition and height for age measurements in your study? I thought height for age measurements are used to measure chronic malnutrition.

- Author response: For this study there were more than one dependent variables. Thus, High-risk fertility behavior among reproductive women were the outcome variables for women. Mentioned as “High-risk fertility behavior is the outcome of interest for women who gave birth, defined as women age at birth less than 18 or above 34 years or birth interval less than 24 months or high birth order were criteria used to define the outcome of the interest.”
- **Secondary outcomes of the study :** Chronic malnutrition like Stunting and Anemia were also outcome variables for children to see any association between chronic malnutrition and High-risk fertility behavior among reproductive age women. Please note that for decision of HFRB and nutritional assessment used was for the recent child and birth. The details about the variables of the study mentioned in the method sections of the study. Page 5-6, Line 101-132.
  Which are the exploratory variables? How were they defined? How were they expressed in the analysis?
- Author response: Corrected in the main document of the manuscript. Mentioned page 5-6, Line 101-132.

Reviewer comments: How was statistical bias avoided? In general, the sub-section: "Variables of the study" in the methods section should be extensively revised. I would strongly recommend extensive grammar and punctuation editing.

- Author response: Some of the strategies used to reduce bias in this study was using standardized definitions for classification of outcomes like HRFB, nutritional status like Anemia, stunting. Regarding the variables of the study we describe in detail in the method section of the manuscript. In addition we tried to address the language error through proof read by all authors.
