# Supplementary material for: Determinants of maternal high-risk fertility behaviors and its correlation with child stunting and anemia in the East Africa region: A pooled analysis of nine East African countries
Source: PLoS One. 2021 Jun 30;16(6):e0253736. doi: 10.1371/journal.pone.0253736 (PMC8244896; doi:10.1371/journal.pone.0253736)
Supplement: Attachment — Submitted filename: Point by point respons_Plos one.docx [file pone.0253736.s002.docx]

**Point by point response**

**Manuscript title: Determinants of maternal high-risk fertility behaviors and its correlation with child stunting and anemia in the East Africa region: A Pooled Analysis of nine East African countries**

**Manuscript ID:** PONE-D-20-27151R1

**Journal – PLOS ONE**

**Dear editor/reviewer**

Dear all,

We would like to thank you for this constructive, building, and improvable comments on this manuscript that would improve the substance and content of the paper. We considered each comment and clarification questions of editors and reviewers on the document thoroughly. Our point-by-point responses for each comment and issues are described in detail on the following pages. Further, the details of changes were shown by track changes in the supplementary document attached.

Koku Sisay Tamirat

On behalf of all authors

**Revise your grammar:**
Line 28, .......child marriage, and a poor health system contributes to high-risk fertility behavior in the East African region.

*Author response: Thanks reviewer for your constructive comments based on your suggestion grammatical errors corrected in the main document. Mentioned in Page 2, Line 28*
Line 39, ……….were common among women who live in rural areas, are unable to access healthcare, have history of abortion, have better economic conditions and had antenatal care follow-up.

*Author response: Thanks for your constructive comments it is already corrected in the main document abstract section. Page 2*
I would recommend you use 'Young maternal age at first birth (<18)" rather than "younger women at first birth".

*Author response: Thanks for your constructive comments it is already corrected in the main document abstract section. Page 2*
Line 115 - 116, what do you mean by ".....to see the relationship between risky behaviors with the child chronic malnutrition and anemia defined as follow," - this is hard to understand. Please revise the grammar.

Author response: *uthor response: Thanks for your constructive comments it is already corrected in the main document as “*Children health outcomes: another objective of this study was to see the association between maternal risky fertility behaviors and chronic malnutrition and anemia in children”.

What is the difference between "Unavoidable risk category" and "No high-risk fertility behavior"?.

Author response: Maternal health outcome: For this study, maternal high-risk fertility behavior was the primary outcome variable which is defined based on several criteria’s as follow;

- High-risk fertility behavior is the outcome of interest for women who gave birth, defined as women age at birth less than 18 or above 34 years or birth interval less than 24 months or high birth order were criteria used to define [16].
- Single high-risk fertility behavior: when a woman reported to had one high-risk fertility behavior the is either younger age less than 18 years, or older age above 34 years, or birth interval less than 24 months, or high-birth order (four and above) [3, 17-19].
- Multiple high-risk fertility behavior: when a woman had a combination of at least two above-mentioned behaviors [3, 17-19]. Unavoidable high-risk fertility behavior is defined as women whose age was between 18 and 34 years and first birth order[16, 17].
- Unavoidable HRFB: when first-order births between ages of 18 and
  34 years in women not amenable to the interventions.
- Not in any high-risk category: when women don’t have any risk fertility behavior
